# Prevalence of *Campylobacter* Species on Cattle Breeding Farms in Zimbabwe

**DOI:** 10.1155/2022/1531483

**Published:** 2022-11-29

**Authors:** Precious Mahlangu, Stephen T. Marambe, Iredale K. Mutengwa, Bamusi Saidi, Pious V. Makaya, John Kagira

**Affiliations:** ^1^Central Veterinary Laboratory, 18 Borrowdale Road, Harare, Zimbabwe; ^2^Department of Animal Sciences, Jomo Kenyatta University of Agriculture and Technology, P.O. Box 62000-00200, Nairobi, Kenya

## Abstract

Infertility remains a challenge in breeding herds in most developing countries. In the current study, 104 penile sheath washes were collected from bulls of different breeds and ages from different cattle breeding farms in Zimbabwe. The samples were submitted to the Central Veterinary Laboratory, Zimbabwe, for screening of *Campylobacter* species using the polymerase chain reaction (PCR). Based on the PCR results, the animal-level prevalence was 25.96% (range 0–73.98%) and all the positive samples came from four (57.14%) of the 7 herds tested. The current study shows that *Campylobacter* spp. could be a causative agent in infertility observed in a number of herds in Zimbabwe. Strategies for treatment and control of campylobacteriosis should be enhanced in the country. More research and surveillance are needed to determine the epidemiology of *Campylobacter* species in Zimbabwean cattle herds.

## 1. Introduction

Campylobacteriosis is an infection caused by the bacteria *Campylobacter* spp. and is associated with a number of disease conditions in cattle, primarily related to poor fertility in breeding cows [1]. *Campylobacter fetus* subspecies *fetus* and *Campylobacter fetus* subspecies *venerealis* are most commonly associated with early embryonic death and abortion [2], the latter of which causes bovine genital campylobacteriosis (BGC). Reproductive diseases of cattle, which result in infertility and abortion, cause significant economic losses [3]. Financial losses arise from extended calving intervals, increased age at first calving, low pregnancy rates, and temporary infertility in bulls [4]. In countries that maintain reproductive management based on natural mating with bulls such as Zimbabwe, mainly in beef herds where natural breeding prevails, the disease may be prevalent. In a study by Swanepoel et al. [5], BGC emerged as one of the most common identified causes of infertility and abortion in Rhodesia (now Zimbabwe). In 1988, a prevalence of 100% was recorded on a farm in Harare, Zimbabwe [6].

In this study, the prevalence of *Campylobacter* spp. in breeding bulls was investigated in samples submitted to the Central Veterinary Laboratory for diagnostic testing.

## 2. Materials and Methods

### 2.1. Study Animals and Diagnostic Tests

The Central Veterinary Laboratory (CVL), Zimbabwe, provided veterinary diagnostic testing services to farmers and public and private veterinary practitioners. The diagnostic tests carried out include tests for bovine sexually transmitted infections (STIs) such as *Campylobacter* spp. One hundred and four bulls of different ages and breeds were sampled from extensive beef production farms between September 2021 and February 2022. The bulls came from a total of seven farms. The farms are located in different provinces of Zimbabwe, that is, Mashonaland East, Harare, and Matabeleland North. Veterinarians submitted samples for different reasons including prebreeding examinations, surveillance, low pregnancy rates in cows, and abortions.

Skilled veterinarians carried out preputial scraping of the bulls to obtain samples for diagnostic testing. The collected washes were put in a 10 ml container with phosphate-buffered saline (PBS-pH 7.0) and sent to the CVL in cooler boxes for analysis within 24 hours.

### 2.2. Sample Analysis

#### 2.2.1. DNA Extraction, Polymerase Chain Reaction, Gel Electrophoresis, and Visualisation

DNA was extracted from the samples using the Zymo Quick DNA Miniprep Plus kit (Zymo, U S A) according to the manufacturer's instructions. The concentration and purity of extracted DNA were checked using a Nanodrop spectrophotometer (Serva electrophoresis, Denmark).

25 *μ*l reaction containing 9.3 *μ*l of RNA-free water, 8.7 *μ*l of the master mix (One Taq 2X Master Mix with standard buffer, New England Biolabs), 1 *μ*l each of *Campylobacter* spp. specific primers (primer sequence F-5′ GGC TGA TCT ACG ATT ACT AGC GAT 3′ and R-3′GCG CGC ATT AGA TAC CCT AGT AGT CC 5′), and 5 *μ*l of the DNA template was used in each PCR tube. Nuclease-free water was used as a negative control. The presence of *Campylobacter* spp. DNA was determined using PCR reaction undertaken as previously described [7], with modifications in the number of cycles, that is, addition of 4 minutes of denaturation at 94°C to cycles 1–5 and reduction of annealing temperature from 37°C to 30°C. Final elongation was carried out at 60°C for 7 minutes instead of 10 minutes.

The PCR test was carried out using a thermocycler (Eppendorf Mastercycler, Nexus Gradient Thermal Cycler).

Gel electrophoresis was carried out using 10 *μ*l PCR products alongside a 100 kb ladder on 2% agarose gel containing a fluorescent dye in 1X TAE buffer at 90 volts for 1 hour. Visualisation of the gel was performed using a gel documentation system (Kodak Gel Logic System 100). The expected band size for *Campylobacter* spp. was 600 base pairs (bp).

### 2.3. Data Analysis

Data were entered into MS Excel (Microsoft, USA), and animal prevalence was determined by calculating the number of positive bulls divided by the total number of sampled bulls. Herd prevalence was calculated by the number of herds, which had at least one bull testing positive for *Campylobacter* spp., divided by the total number of herds tested.

## 3. Results

### 3.1. Prevalence of *Campylobacter* spp

The animal-level prevalence of *Campylobacter* spp. was 25.96%, and all the positive samples came from four (57.14%) of the 7 herds. Only three (42.9%) herds out of 7 herds had all the bulls testing negative for *Campylobacter* spp. (Table 1).

## 4. Discussion

The cattle population in Zimbabwe is estimated to be 5.2 million (Director, Division of Veterinary Technical Services). In recent years, farmers have lost cattle due to drought and diseases. The current thrust of the Zimbabwean government is to replenish and increase the national herd [8]. One of the ways the government has been advocating for this increase is through improvement of cattle breeding through natural breeding and artificial insemination. However, these efforts are being hampered by reproductive diseases such as those caused by *Campylobacter* spp. The disease is known to hamper economic growth where farmers incur huge losses [9]. The literature on the prevalence of *Campylobacter* spp. in Southern African and other developing countries is scarce.

The current prevalence and impact of campylobacteriosis in Zimbabwe are unknown. The last known study on prevalence of *Campylobacter fetus* subspecies *venerealis* was performed in 1989 [6], where 100% of the bulls had BGC. In recent years, veterinarians and farmers have reported existence of infertility problems in cows, and this is suspected to be due to sexually transmitted infections such as BGC. Furthermore, in the past years, movement of cattle increased due to the land reform program, resettlements, and social and cultural reasons [10]. Premovement permits were focusing mainly on foot and mouth disease, while production and reproductive diseases were not being checked. This could spread diseases such as campylobacteriosis across the country.

In this study, only 3 herds out of 7 had all bulls testing negative for *Campylobacter* spp. The high prevalence of *Campylobacter* spp. infection noted in this study warrants further research on campylobacteriosis in Zimbabwe. It is important to note that the positive *Campylobacter* spp. samples identified in the CVL laboratory were the first in over ten years (Personal Communication, Technologist-in-charge, CVL, Zimbabwe). In the past decade, farmers and animal movement regulatory authorities did not focus much on bovine sexually transmitted infections, and this may have resulted in the introduction of the infection into herds unknowingly through cattle movement, loaning, and sharing of bulls [9]. The difference in prevalence across the herds in the country could be due to management systems. Previous studies have shown that prevalence of *Campylobacter* spp. is affected by climate change, locations, animal breeds, and age.

As STIs decrease productivity of cattle by inducing reproductive losses, reduced conception rates, extended calving seasons, and increased costs, replacing bulls and preventing loss of genetic potential due to culling the disease are of great economic importance. A comprehensive study should be carried out in Zimbabwe to determine the epidemiology of *Campylobacter* spp. with the aim of developing intervention to reduce such losses.

## 5. Conclusion and Recommendation

The study reports a high prevalence of *Campylobacter* spp. in bull herds in Zimbabwe. There is a need for further surveillance of *Campylobacter* spp. in the country using advanced molecular techniques, which will be able to elucidate the subspecies responsible. Veterinary authorities should enforce strategies which can minimize the spread of cattle STIs in the country.

## Figures and Tables

**Table 1:** Results of samples tested for *Campylobacter* spp. in the study.

Herd	Number of *Campylobacter* spp. positive samples	Number of *Campylobacter* spp. negative samples	Total number of samples submitted	Animal prevalence (%)
1	4	13	17	30.77
2	0	8	8	0
3	8	11	19	42.11
4	0	20	20	0
5	0	2	2	0
6	1	18	19	0.05
7	14	5	19	73.68
Total	27	77	104	25.96

## Data Availability

Raw data supporting this article are available from the corresponding author upon request.

## References

[1] Mshelia G. D., Amin J. D., Woldehiwet Z., Murray R. D., Egwu G. O. (2010). Epidemiology of bovine venereal campylobacteriosis: geographic distribution and recent advances in molecular diagnostic techniques. *Reproduction in Domestic Animals*.

[2] BonDurant R. H. (2005). Venereal diseases of cattle: natural history, diagnosis, and the role of vaccines in their control. *Veterinary Clinics of North America: Food Animal Practice*.

[3] Weersink A., VanLeeuwen J. A., Chi J., Keefe G. P. (2002). Direct production losses and treatment costs due to four dairy cattle diseases. *Advances in Dairy Technology*.

[4] Michi A. N., Favetto P. H., Kastelic J., Cobo E. R. (2016). A review of sexually transmitted bovine trichomoniasis and campylobacteriosis affecting cattle reproductive health. *Theriogenology*.

[5] Swanepoel R., Blackburn N. K., Lander K. P., Vickers D. B., Lewis A. R. (1975). An investigation of infectious infertility and abortion of cattle. *Rhodesian Vet J*.

[6] Woldehiwet Z., Odiawo G. O., Pawadiwa A. (1989). Isolation of *Campylobacter fetus* subsp. *venerealis* from bulls in a beef herd in Harare. *Zimbabwe Veterinary Journal*.

[7] Blom K., Patton C. M., Nicholson M. A., Swaminathan B. (1995). Identification of Campylobacter fetus by PCR-DNA probe method. *Journal of Clinical Microbiology*.

[8] Zimbabwe National Development Strategy (2021).

[9] Van Bergen M. A. P., Linnane S., Van Putten J. P. M., Wagenaar J. A. (2005). Detección e identificación de Campylobacter fetus subesp. venerealis en el mundo. *Revue Scientifique et Technique de l’OIE*.

[10] Department of Veterinary Services (2020). *Zimbabwe Annual Report*.

